# Maternal Preeclampsia Is Associated With Reduced Adolescent Offspring Hip BMD in a UK Population‐Based Birth Cohort

**DOI:** 10.1002/jbmr.2506

**Published:** 2015-05-14

**Authors:** Kimberly Hannam, Debbie A Lawlor, Jon H Tobias

**Affiliations:** ^1^ Musculoskeletal Research UnitUniversity of BristolBristolUK; ^2^ MRC Integrative Epidemiology UnitUniversity of BristolBristolUK; ^3^ School of Social and Community Medicine, University of BristolBristolUK

**Keywords:** GESTATIONAL HYPERTENSION, DXA, ALSPAC

## Abstract

A suboptimal intrauterine environment has been postulated to have adverse long‐term health effects, including an increased risk of osteoporosis. Because preeclampsia (PE) and to a lesser extent gestational hypertension (GH) are associated with impaired placental function, we postulated that these represent hitherto unrecognized risk factors for reduced bone mineral density (BMD) of the offspring. The objective of this study was to investigate if exposure to PE or GH in utero is associated with BMD of the offspring as measured in late adolescence. Mother‐offspring pairs from the UK population‐based cohort study, Avon Longitudinal Study of Parents and Children (ALSPAC), were investigated (*n* = 3088 with relevant data). Multivariable linear regression was used to examine associations between PE/GH and total body, spine, and total hip BMD at age 17 years. Of the 3088 mother‐offspring pairs, 2% (*n* = 60) of the mothers fulfilled criteria for PE and 14% (*n* = 416) for GH. In confounder‐adjusted analyses (ie, age of scan, gender, maternal factors, including BMI, offspring height, fat mass, and lean mass), PE was negatively associated with BMD at the hip (SD difference –0.30; 95%CI, –0.50 to –0.10). This association was not attenuated by further adjustment for gestational age and birth weight, which were hypothesized to be on the causal pathway. There was also weak evidence for a negative association between PE and total body BMD (SD difference –0.17; 95% CI, –0.36 to 0.02), whereas no relationship was evident at the spine (SD difference –0.11; 95% CI, –0.30 to 0.09). In contrast, a positive association of GH with offspring total body, hip, and spine BMD attenuated to the null with adjustment for confounders, in particular confounding via the maternal and offspring adiposity/size and the link between the two. Modest negative associations from exposure to PE, but not GH may represent a hitherto unrecognized risk factor for low BMD. Further exploration of the causal relationship of the in utero environment on subsequent offspring bone health is required. © 2015 The Authors. *Journal of Bone and Mineral Research* published by Wiley Periodicals, Inc. on behalf of the American Society for Bone and Mineral Research.

## Introduction

Nutritional deprivation of the fetus during pregnancy may increase the risk of developing a range of chronic diseases in later life, including osteoporosis.^1^ Adverse environmental conditions in utero are proposed to affect the trajectory of subsequent skeletal growth and development, resulting in suboptimal bone structure and an increased risk of osteoporotic fracture in later life. Birth weight (BW) has been widely used as a proxy measure for nutritional status during pregnancy in studies examining relationships between adverse exposures in utero and bone outcomes in later life.^2^, ^3^ Other factors studied as proxies for intrauterine nutritional deficiency include vitamin D status,^4^ maternal diet,^5^, ^6^ and age.^7^

Theoretically, hypertensive disorders of pregnancy (HDP), which are related to vascular endothelial dysfunction, poor placentation, and small‐for‐gestational‐age, may also impair long‐term bone outcomes in the fetus, but this has been little studied to date. Preeclampsia (PE) is a systemic condition that affects 3% to 7% of all pregnancies and is characterized by hypertension and proteinuria, whereas gestational hypertension (GH) affects 6% to 17% of pregnancies and is characterized by hypertension without proteinuria.^8^, ^9^ The first stage of PE in early pregnancy, which is asymptomatic, is characterized by alterations in placental function and development resulting in reduced placental perfusion. The second stage is the maternal syndrome that occurs in later pregnancy where clinically detectable signs and symptoms of preeclampsia are present due to increased vascular permeability, glomerular endotheliosis, and systemic inflammatory response.^10^, ^11^ PE and GH are both associated with adverse health outcomes for the mother and offspring in utero, during the perinatal period and in later life.^8^, ^12^, ^13^ For example, PE is associated with an increased risk in the offspring in later life of higher blood pressure,^14^, ^15^ stroke,^16^ and lower cognitive ability and decline up to old age.^17^, ^18^

To our knowledge, only one previously published paper has examined the associations between HDP and offspring BMD.^19^ That study included 144 very low BW (32 with PE) and 139 term offspring (11 with PE) and measured BMD at 18.5 to 27.1 years. Perhaps surprisingly, they reported that offspring exposed to PE, born at very low BW and at term, had a higher BMD than those not exposed. In the present study, we aimed to explore these associations further in a large general population based cohort, by linking the occurrence of PE in pregnant mothers from the Avon Longitudinal Study of Parents and Children (ALSPAC) with BMD assessed in offspring at age 17 years. In addition, we aimed to extend these analyses by studying whether equivalent associations are present between GH and offspring BMD.

## Subjects and Methods

### Study population

ALSPAC is a prospective population‐based cohort located in the Bristol area in the UK (www.alspac.bris.ac.uk) that investigates genetic, environmental, and social factors on health throughout the life course. The cohort has been explained in detail elsewhere.^20^, ^21^ Briefly, all pregnant women resident in the defined area within South West England with an expected delivery date between April 1, 1991, and December 31, 1992, were eligible for recruitment and 14,541 were subsequently enrolled. ALSPAC has collected maternal, paternal, and offspring data through linkage to computerized records, postal questionnaires, and research clinics. The present study is based on data collected from obstetric medical notes, antenatal and postnatal questionnaires, and the “@17” adolescence research clinic. Ethical approval for this study was obtained from the ALSPAC Law and Ethics Committee and the Local Research Ethics Committee. Written informed consent was provided by all parents, and young people provided written assent.

### Maternal pregnancy data

Obstetric medical record details were abstracted by six trained midwives. Measurements included all systolic blood pressure (SBP) and diastolic blood pressure (DBP) readings, proteinuria measurements, and the corresponding gestational age (GA) and date. PE and GH were identified based on the criteria from the International Society for the Study of Hypertension in Pregnancy.^22^ Using these guidelines, the following definitions were applied: PE was defined as a SPB >139 mmHg or a DPB >89 mmHg, measured on at least two occasions after 20 weeks of gestation with proteinuria at the same time, diagnosed if the protein reading on dipstick testing (Albustix; Ames Company, Elkhart, IN, USA) was ≥1 (30 mg/dL). Those women with preexisting hypertension and those with GH were not included in the PE group. GH was defined as the same pattern in BP measurements but without proteinuria, again excluding those women with preexisting hypertension.^22^ All ALSPAC mothers were characterized into one of three mutually exclusive gestational hypertensive disorder categories: PE, GH, or no HDP. The median number of BP measurements recorded and extracted from participant medical records was 14 (IQR, 11 to 16) and a median of 11 (IQR, 10 to 14) urine measurements.

### Offspring outcome measurements

All offspring who attended the ALSPAC research clinic at 17 years old were offered a whole‐body and hip dual‐energy X‐ray absorptiometry (DXA) scan using a GE Lunar Prodigy (Madison, Wisconsin), providing total (body less head), spine subregion, and hip BMD (g/cm^2^) measurements. Whole‐body DXA also provided total body lean mass (kg) and fat mass (kg) measurements. Offspring height was measured using a Harpenden stadiometer (Holtain Ltd., Crymych, UK), and weight was measured to the nearest 50 g using Tanita weighing scales (Tanita UK Ltd, Uxbridge, UK). Error codes were generated for each DXA variable obtained (movement, artifacts, and positioning errors).

### Other covariate data

**Figure 1:**
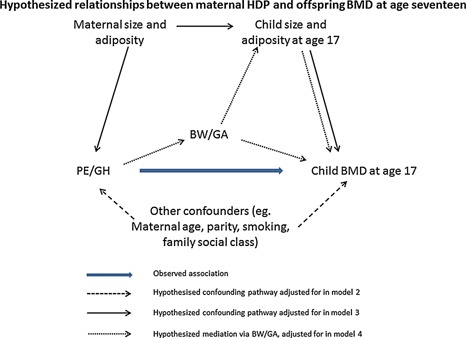
Childhood size and adiposity could be part of a confounding or mediation pathway. The confounding pathway would generate a positive association of PE/GH with BMD. The mediation pathway (because PE/GH is inversely associated with BW, which is positively associated with child size and adiposity) would generate an inverse relationship. HDP = hypertensive disorders of pregnancy; PE = preeclampsia; GH = gestational hypertension; BW = birth weight; GA = gestational age; BMD = bone mineral density.

Age at scan, gender, maternal age, maternal smoking, maternal BMI, parity and offspring height, fat mass, and lean mass were considered as potential confounders. BW and GA were considered as potential mediators. Although offspring fat and lean mass at the time of BMD scan, by definition, occur after exposure to PE and GH, we considered them to be part of a confounding path from maternal BMI (which is a known risk factor for PE and GH) via offspring adiposity (assessed by fat and lean mass and which are influenced by maternal size and her genetic and lifestyle characteristics that are shared with her offspring and influence adiposity) to offspring BMD (see Fig. 1). Maternal smoking data was collected throughout pregnancy. For this study, data were used from the questionnaire administered at 32 weeks, which categorized self‐reported smoking into none, 1 to 9 cigarettes per day, 10 to 19 cigarettes per day, and ≥20 cigarettes per day. The variable was collapsed to become a yes/no variable of smoking in pregnancy. At the time of recruitment, participants were asked to self‐report their prepregnancy height and weight, from which maternal prepregnancy BMI was calculated (weight (kg)/height squared (m^2^)). A very strong correlation was found between self‐reported prepregnancy weight and measured weight at the first antenatal clinic (*r* = 0.95; *p* < 0.01). Maternal BMI was categorized into four standardized categories: underweight (<18.5 kg/m^2^), normal (18.5 to 24.9 kg/m^2^), overweight (25 to 29.9 kg/m^2^), and obese (≥30 kg/m^2^). Social class group of the parents and child was determined based on reported highest parental occupation from questionnaire responses. The groups were generated using the 1991 British Office of Population and Census Statistics Classification, with groups ranging from class I (professional/management) to V (unskilled manual workers). Parity was determined through self‐reported questionnaires and was categorized into five categories: 0 previous pregnancies; 1 previous pregnancy, 2 previous pregnancies, 3 previous pregnancies, and ≥4 previous pregnancies. BW in grams was obtained from birth records, and GA was calculated based on last menstrual period (recorded in maternal medical records) and actual date of delivery. Preterm birth (PTB) was defined as less than 37 weeks completed gestation and low BW (LBW) was defined as BW <2500 g.

### Statistical analyses

All analyses were performed in STATA, version 13. For all descriptive analyses arithmetic means are presented. Multivariable linear regression analyses were used to determine associations between mothers with PE and GH in comparison with the reference category of no HDP with offspring outcome measures. DXA‐determined fat mass was positively skewed and thus was log‐transformed to produce an approximate normal distribution. All outcome variables were standardized for the regression analyses, thus the beta coefficients represent a mean standard deviation (SD) difference in each of the DXA measured variables for each comparison of HDP groups. Multivariable linear regression analyses were performed to explore the associations between HDP classification with total body, total hip, and spine subregion BMD at 17 years old. We hypothesized that an observed association between HDP and offspring BMD could occur because of: (1) confounding by maternal/ familial characteristics, such as socioeconomic position; (2) confounding by adiposity and shared genetic/familial characteristics that provide a confounding pathway involving maternal adiposity and child adiposity and size^23^; or (3) a causal effect, with this possibly being mediated via the effect of HDP on fetal growth and development (represented by BW and GA) and of those on subsequent BMD. Figure 1 shows these hypothesized pathways; we used this figure to develop the analytical models that we would undertake. In the basic model (model 1) we adjusted for gender and age at DXA scan. In model 2, we additionally adjusted for potential confounding by maternal age, parity, smoking during pregnancy, and familial social class. To explore the role of additional confounding by a pathway involving maternal and child adiposity/size, in model 3 we additionally adjusted for prepregnancy BMI and offspring height, fat mass, and lean mass at age 17 years. Finally, in model 4 we also adjusted for potential mediation by GA and BW.

## Results

**Figure 2:**
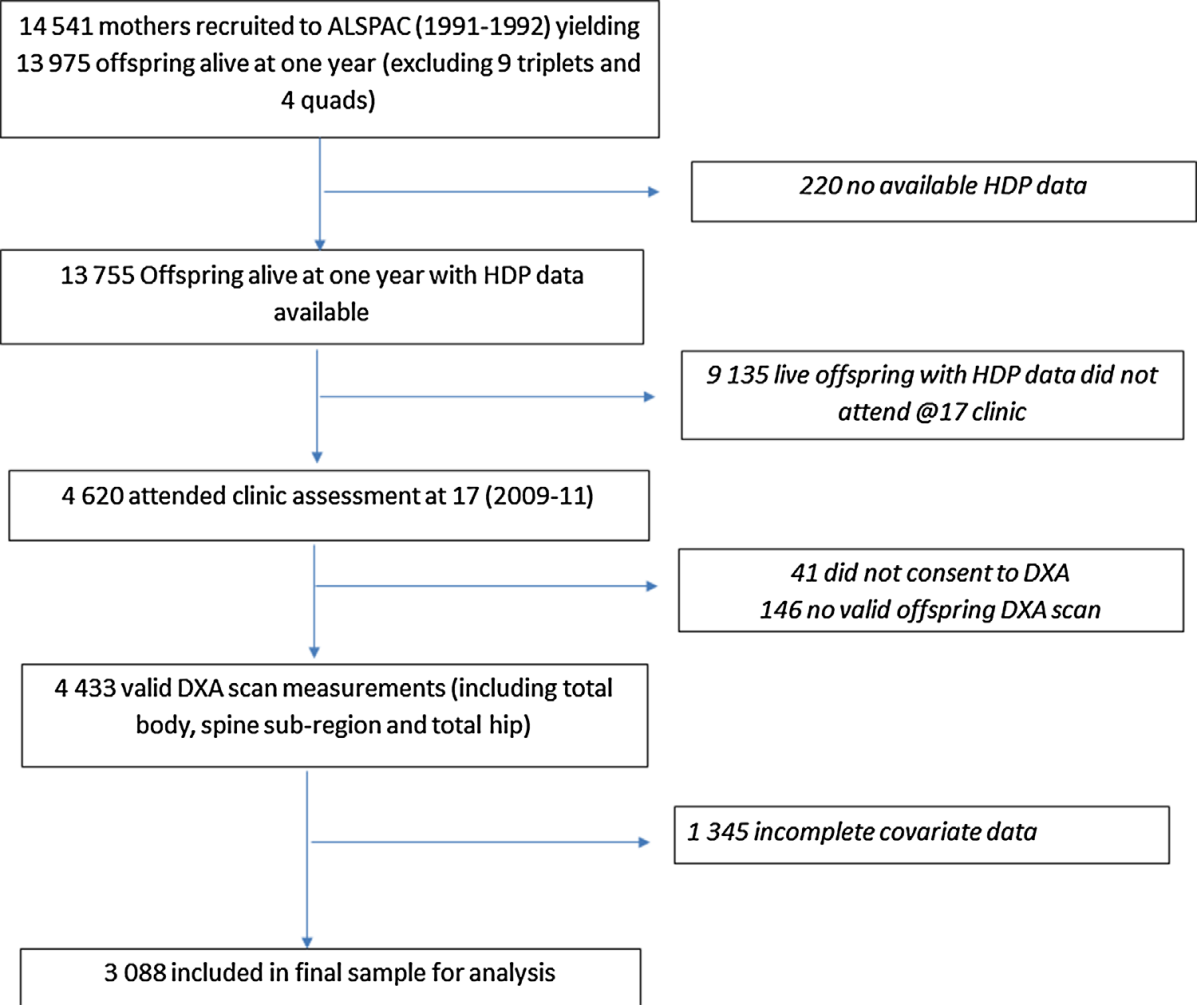
Flow diagram of ALSPAC participants. ALSPAC = ; HDP = hypertensive disorders of pregnancy; DXA = dual‐energy X‐ray absorptiometry.

A total of 13,755 ALSPAC mothers had data on HDP and offspring alive at 1 year (excluding nine triplets and four quadruplets), of whom 4620 attended the 17+ research clinic, yielding 3088 mother‐offspring pairs with complete information about all relevant DXA outcomes and other covariates, forming the basis for the present analysis (Fig. 2). Mothers included in these 3088 mother‐offspring pairs, compared to all 13,755 mothers with HDP data, were older, of higher social class, less likely to smoke, and less likely to be overweight or obese, but had a similar prevalence of HDP, and birth/infant outcomes were similar (Supporting Table 1). We found no strong evidence of differences in any associations of PE or GH with BMD, between females and males (all *p* values for interaction of exposure with gender ≥0.29) and thus results are presented with females and males combined.

### Participant characteristics

Table 1 shows the maternal characteristics from the eligible participants included in our analyses. Of the 3088 eligible mother‐offspring pairs, 60 (2%) had PE, 416 (13%) had GH, and 2612 (85%) had no HDP. Mothers who had PE were more likely to be over 35 years of age, nonsmokers, nulliparous, in a lower social class, and have a greater prepregnancy BMI. Mothers with GH were more likely to be nonsmokers and nulliparous or have had at least four previous deliveries, and a greater prepregnancy BMI.

**Table 1:** Maternal Characteristics by Categories of Hypertensive Disorder of Pregnancy

	Preeclampsia		Gestational hypertension		No HDP		Total	
	*n*	%	*n*	%	*n*	%	*n*	%
Total	60	1.9	416	13.5	2612	84.6	3088	100
Maternal age								
15–19 years	2	3.3	5	1.2	29	1.1	36	1.2
20–24 years	9	15.0	68	16.4	290	11.1	367	11.9
25–29 years	14	23.3	142	34.1	1009	38.6	1165	37.7
30–34 years	23	38.3	136	32.7	944	36.1	1101	35.7
35–39 years	12	20.0	54	13.0	302	11.6	368	11.9
>40 years	0	0.0	11	2.6	40	1.5	51	1.7
Maternal smoking in pregnancy								
Yes	4	6.7	36	8.7	311	11.9	351	11.4
No	56	93.3	380	91.4	2301	88.1	2737	88.6
Parity								
0	44	73.3	272	65.4	1285	49.2	1601	51.9
1	9	15.0	104	25.0	955	36.6	1068	34.6
2	7	11.7	30	7.2	301	11.5	338	11.0
3	0	0.0	8	1.9	61	2.3	69	2.2
4+	0	0.0	2	0.5	10	0.4	12	0.4
Social class								
I	3	5.0	32	7.7	223	8.5	258	8.4
II	21	35.0	150	36.1	957	36.6	1128	36.5
III (non‐manual)	25	41.7	170	40.9	1040	39.8	1235	40.0
III (manual)	4	6.7	32	7.7	154	5.9	190	6.2
IV	7	11.7	28	6.7	205	7.9	240	7.8
V	0	0.0	4	1.0	33	1.3	37	1.2
Maternal BMI								
Underweight (<18.5 kg/m^2^)	1	1.7	6	1.4	131	5.0	138	4.5
Normal (18.5–24.9 kg/m^2^)	37	61.7	291	70.0	2095	80.2	2423	78.5
Overweight (25–29.9 kg/m^2^)	10	16.7	91	21.9	299	11.5	400	13.0
Obese (≥30 kg/m^2^)	12	20.0	28	6.7	87	3.3	127	4.1

Maternal characteristics for 3088 maternal‐offspring pairs included in the study.

HDP = hypertensive disorder of pregnancy.

Offspring characteristics according to HDP status are shown in Table 2. Mothers with PE were much more likely to have a preterm and low BW infant compared to mothers with GH no HDP. Offspring of those with PE or GH were on average heavier and had greater fat and lean mass compared to those without HDP. Compared to offspring of women with no HDP, offspring to mothers with PE had similar mean total and spine BMD, whereas mean total hip BMD appeared lower. Mean total, spine, and hip BMD appeared to be higher in offspring of women with GH compared to those of women with no HDP.

**Table 2:** Offspring Characteristics by Maternal Categories of HDP

	Preeclampsia		Gestational hypertension		No HDP	
	*n*		*n*		*n*	
GA (weeks), mean ± SD	60	38.1 ± 2.3	416	40.0 ± 1.8	2612	39.5 ± 1.7
PTB (%)	13	21.7	22	5.3	125	4.8
Birth weight (g), mean ± SD	60	3092 ± 774	416	3406 ± 594	2612	3418 ± 506
LBW (%)	13	21.7	28	6.7	105	4.0
Age at scan (years), mean ± SD	60	17.9 ± 0.5	416	17.8 ± 0.4	2612	17.8 ± 0.4
Male gender (%)	33	55.0	208	50.0	1175	45.0
Height (cm), mean ± SD	60	171.6 ± 9.4	415	172.8 ± 9.0	2601	171.3 ± 9.0
Weight (kg), mean ± SD	60	68.4 ± 12.2	415	68.0 ± 12.6	2602	64.9 ± 11.1
Fat mass (kg), mean ± SD	60	18.82 ± 11.53	416	17.79 ± 10.04	2612	16.50 ± 8.43
Lean mass (kg), mean ± SD	60	46.44 ± 9.70	416	46.98 ± 9.98	2612	45.38 ± 9.75
Total body BMD (g/cm^2^), mean ± SD	60	1.17 ± 0.10	416	1.19 ± 0.93	2612	1.17 ± 0.09
Spine BMD (g/cm^2^), mean ± SD	60	1.11 ± 0.18	416	1.13 ± 0.15	2612	1.11 ± 0.14
Total hip BMD (g/cm^2^), mean ± SD	60	1.07 ± 0.15	416	1.13 ± 0.16	2612	1.11 ± 0.15

Offspring characteristics for 3088 maternal‐offspring pairs included in the study. Height, weight, fat mass, lean mass, and BMD measurements were obtained at offspring age 17 years. Values are % or mean ± SD, as indicated.

HDP = hypertensive disorder of pregnancy; GA = gestational age; PTB = preterm birth; LBW = low birth weight; BMD = bone mineral density.

### Association of PE with offspring BMD

**Figure 3:**
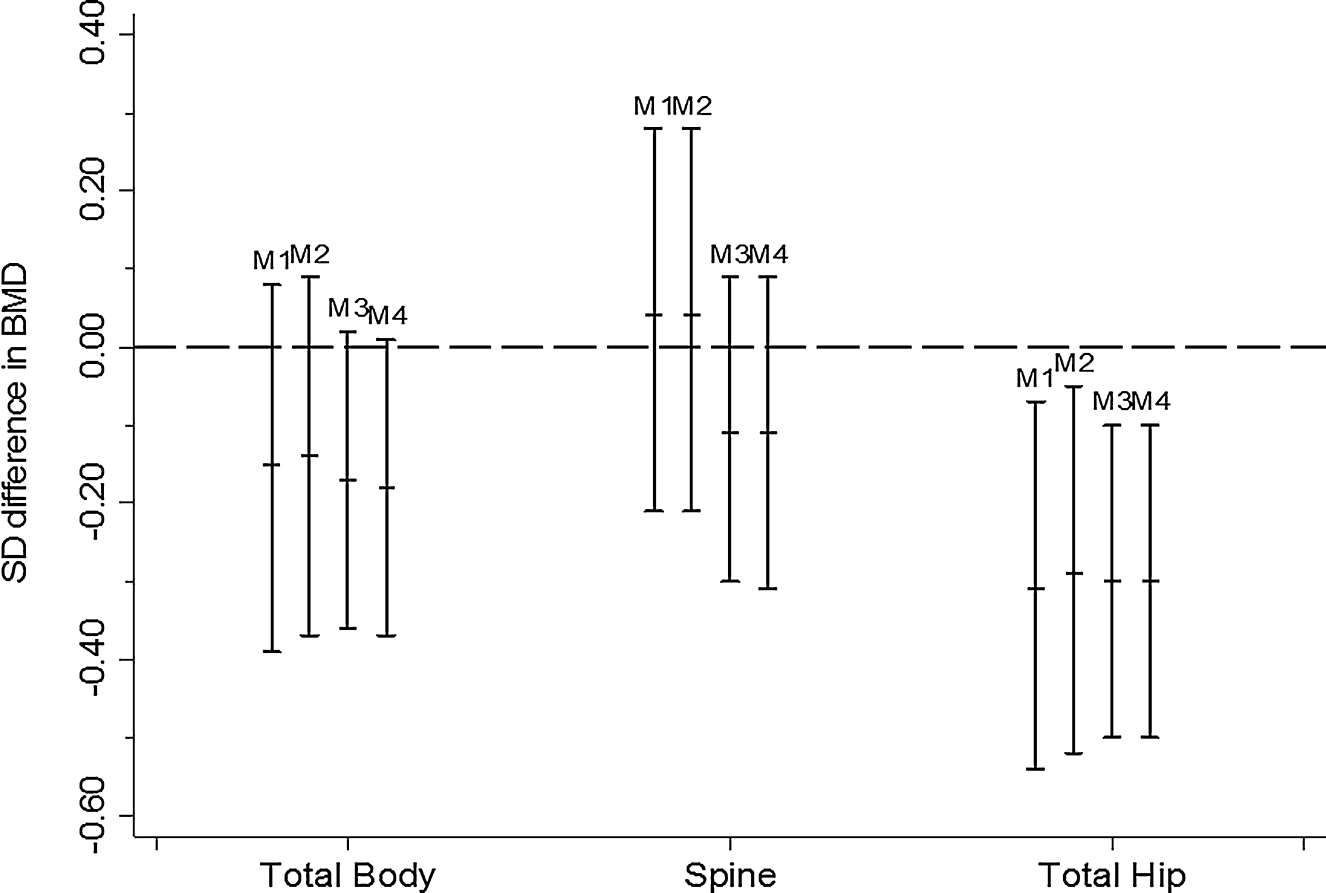
Associations between preeclampsia and BMD measured at 17 years for 3088 maternal‐offspring pairs included in the study (whiskers indicating 95% CIs). Model 1: age at scan and gender; Model 2: 1 + maternal smoking, socioeconomic status, maternal age, parity; Model 3: 2 + maternal BMI, offspring fat mass, lean mass and height; Model 4: 3 + birth weight and gestational age. BMD = bone mineral density.

Total hip BMD was lower in offspring who were exposed to PE compared to those who were not, with adjustment for gender and age (SD difference –0.31; 95% CI, –0.54 to –0.07; *p* = 0.01) (Fig. 3; Supporting Table 2). There was weak evidence of a small negative association between PE and total body BMD (SD difference –0.15; 95% CI, –0.39 to 0.08; *p* = 0.19), whereas no association was observed for lumbar spine BMD. These inverse associations were not markedly altered by adjustment for confounders (models 2 and 3). Nor did they appear to be mediated by BWand GA (model 4).

### GH versus offspring BMD

**Figure 4:**
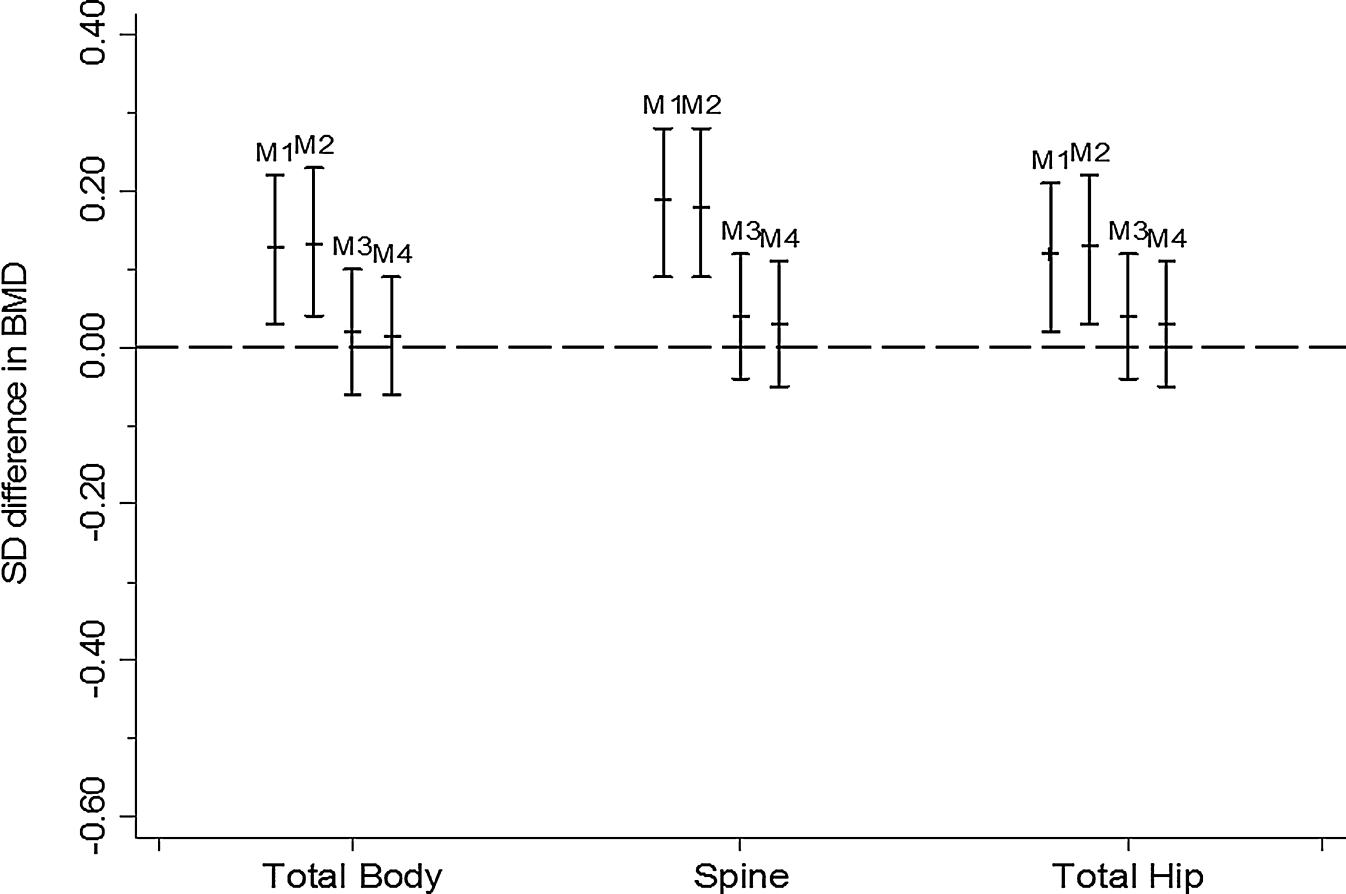
Associations between gestational hypertension and BMD measured at 17 years for 3088 maternal‐offspring pairs included in the study (whiskers indicating 95% confidence intervals). Model 1: age at scan and gender; Model 2: 1 + maternal smoking, socioeconomic status, maternal age, parity; Model 3: 2 + maternal BMI, offspring fat mass, lean mass and height; Model 4: 3 + birth weight and gestational age. BMD = bone mineral density.

Total BMD at all three sites was greater in offspring who had been exposed to GH compared to those who were not, with adjustment for gender and age (total body BMD: SD difference 0.13; 95% CI, 0.03 to 0.22; *p* = 0.007; spine BMD: SD difference 0.19; 95% CI, 0.09 to 0.28, *p* < 0.001; total hip BMD: SD difference 0.13; 95% CI, 0.03 to 0.22; *p* = 0.009) (Fig. 4; Supporting Table 2). However, these positive associations appeared to be explained by the maternal BMI–offspring adiposity/size confounding pathway (model 3).

## Discussion

We examined the relationship between HDP and BMD of the offspring in 3088 mother‐offspring pairs from a population‐based cohort. We found that hip BMD in offspring of mothers who developed PE during pregnancy was lower by approximately one‐third of an SD, equating to –0.05 g/cm^2^, as measured at age 17 years. In contrast, no association was observed between maternal PE and BMD of the spine subregion. Because the relationship between BMD and fracture risk is in part site‐specific,^24^ it is tempting to speculate that, should this BMD deficit persist into later life, it may ultimately result in a greater fracture risk in those born to mothers with PE, particularly at the hip. However, the magnitude of the association is relatively weak and these observational data cannot prove causality. In contrast, BMD of offspring born to mothers with GH appeared to be greater at all sites, namely hip, total body, and spine subregion, but these positive associations appeared to be due to confounding.

To our knowledge only one previous study has examined the association of HDP with offspring BMD in adolescence/early adulthood.^19^ In that study participants from the Helsinki Study of Very Low Birthweight, infants born preterm with very low BW (<1500 g) between 1978 and 1985, who survived to 2004 (when aged 18.5 to 27.1 years) were invited for examination together with a 1:1 matched control who was an adult of the same sex born around the same time in the same hospital but who was term and not small‐for‐gestational‐age. A total of 144 (32 of whom had PE) very low BW adults and 139 controls (11 with PE) were examined and in each group the association of HDP (defined in a similar way to our definition) with DXA BMD was examined. The focus was primarily on PE, and contrary to our findings, the authors found exposure to PE (compared with no HDP) was associated with higher spine, hip, and total body BMD in both the very low BW group and the control group (though in the latter the positive association with lumbar spine BMD did not reach conventional levels of statistical significance). In both groups positive associations remained with adjustment for a wide range of potential confounders. There were too few participants with GH to look at that as a separate exposure, nonetheless the associations with PE clearly differ from our findings of an inverse association. The association in very low BW preterm survivors is not necessarily a sensible comparison with our general population group; not least because only 30% of the original cohort survived and attended the adult follow‐up. However, the control group might be considered to be broadly consistent with our study participants. Thus, the difference between those two groups is harder to explain. Our sample size was larger, which makes it less likely to be a chance finding, but we cannot exclude the possibility (given there are just these two studies) that both represent chance findings around an overall null association.

Nutritional deficiency in utero has been suggested to affect subsequent bone health through fetal programming of numerous metabolic and endocrine systems that regulate skeletal growth.^1^, ^3^ As well as reducing GA,^8^ partly due to iatrogenic deliveries that often occur in PE pregnancies, PE is associated with lower BW due to reduced placental size and placental insufficiency.^8^ Placental calcium transport occurs in the syncytiotrophoblast of the placenta,^25^ where there is evidence that increased apoptosis occurs during PE.^26^ The majority of maternofetal transfer of calcium, phosphorus and magnesium occurs after 24 weeks of gestation and the predominant period for fetal bone development is during the third trimester, consequently GA is also a strong determinant of BMD at birth.^1^ PTB and LBW have been associated with compromised BMD in the neonatal and early childhood period.^27^ Preterm infants are also exposed to postnatal factors that contribute further to reduced bone development, including medication usage, immobilization, and nutrition problems.^28^ However, though LBW and GA are potential mediators of the observed association between PE and BMD, this did not appear to be the case given that additional adjustment for BW and GA had little effect on this relationship.

### Strengths and limitations

The main strength of this study is the large sample size, particularly in comparison to the only previous study investigating the relationship between HDP and BMD.^19^ The ALSPAC cohort has comprehensive maternal and offspring covariate data to enable selection of adjustments to be made without being restricted by data availability. This study benefits from having a particularly robust way to classify GH and PE in pregnancy (using trained midwives to extract all BP and proteinuria measurements taken during pregnancy from obstetric notes), which reduces the risk of misclassification of the exposure. This study was able to accurately adjust for offspring body composition measures through adjustments of fat mass and lean mass.

One of the limitations to this study is the inevitable attrition that occurs in a longitudinal prospective cohort study. Although the subgroup of ALSPAC participants who were not lost to follow‐up and had complete covariate data were generally quite similar to the cohort as a whole, they tended to be older, of a higher socio economic position, less likely to smoke, and less likely to be overweight or obese. However, in a previous study using data from the ALSPAC cohort we have shown that this attrition would generally result in underestimation of associations.^29^ Over 95% of ALSPAC participants are of white European origin and our findings might not necessarily be generalizable to other populations. In light of the observational nature of this study we are unable to infer a causal relationship between PE and BMD; eg, shared factors for a suboptimal intrauterine environment such as poor diet during pregnancy or stress may have contributed to the observed associations. In further analyses examining the potential confounding effects of maternal calcium supplementation, little change in effect sizes between PE and BMD were observed when we adjusted for consumption of calcium supplements (Supporting Table 3). This study investigated offspring BMD at just one time point during adolescence. We were unable to investigate if the observed lower BMD associated with PE within the ALSPAC cohort persists throughout the adult life course ultimately resulting in an increased risk of osteoporosis. DXA data from the ALSPAC offspring collected before 17 years of age were not included in the main results of this study. This was because of a particular interest on hip DXA, which was not measured at the 9‐year follow‐up and because we felt that interpretation of the 13‐year results would be complicated by the possible impact of puberty on associations. It was therefore decided a priori that the latest age of assessment (at 17 years) would be the sole outcome assessed. This was also influenced by our consideration that of the three ages where BMD has been measured in ALSPAC the assessment at age 17 years is likely to be most clinically relevant in terms of longer‐term effects and is also similar to participant ages in the one previous study examining this association. Additional analyses of the association of HDP with total body and spine DXA data collected at age 9 years showed no strong evidence that either PE or GH was associated with total body or lumbar spine analyses at age 9 years (Supporting Table 4). Spinal BMD measures were obtained from subregional analysis of total body DXA scans, which is less accurate than the conventional method based on separate lumbar spine scans. Finally, we acknowledge that in our analyses we have assumed that offspring adiposity and size are part of a confounding factor, but it is possible that they mediate some of the relationship of HDP with offspring BMD; eg, via the relationship of BW to later offspring size (as shown in Fig. 1). For the inverse association of PE with offspring hip BMD, the point estimate remained essentially unchanged across all four models, supporting our conclusion that this inverse association did not appear to be explained by confounding or mediation (for the characteristics in our hypothesized paths). The positive associations of GH with total and site‐specific BMD attenuated to the null with adjustment for maternal BMI and offspring height, fat mass, and lean mass. However, because we have previously shown no association of GH with BW,^23^ and other studies that find an association find an inverse one, a mediation pathway from GH via BW to subsequent offspring adiposity and size would generate an inverse association, not the positive association that we have observed. We therefore do not think that the attenuation of the observed positive GH‐offspring BMD association represents overadjustment for characteristics that are part of a causal pathway.

## Conclusions

We found that exposure to PE during pregnancy is associated with modestly lower adult offspring hip BMD at age 17 years, whereas our results suggest no association of GH with BMD once confounders have been accounted for. In light of these findings, exposure to PE, but not GH, may represent a hitherto unrecognized risk factor for low BMD, and possibly osteoporotic fracture.

## Disclosures

All authors state that they have no conflicts of interest.

## Supporting information

Supporting Information.
