# Supplementary material for: Maternal Preeclampsia Is Associated With Reduced Adolescent Offspring Hip BMD in a UK Population‐Based Birth Cohort
Source: J Bone Miner Res. 2015 May 14;30(9):1684–91. doi: 10.1002/jbmr.2506 (PMC4540657; doi:10.1002/jbmr.2506)
Supplement: Supplementary file 1 — Supporting Information. [file JBMR-30-1684-s001.doc]

**Supporting Table** 1: Maternal and offspring characteristics in the final included sample in this study compared to all ALSPAC live offspring with available HDP data.

| **Descriptive characteristics** | **Total included in the study** | | **Total live offspring with HDP data** | |
| --- | --- | --- | --- | --- |
|  | **n** | **%/mean(SD)** | **n** | **%/mean(SD)** |
| ***Total*** | ***3088*** |  | ***13 755*** |  |
| **HDP** |  |  |  |  |
| Preeclampsia | 60 | 1.94 | 299 | 2.17 |
| Gestational hypertension | 416 | 13.47 | 1 970 | 14.32 |
| No HDP | 2 612 | 84.59 | 11 486 | 83.50 |
| **Maternal age** |  |  |  |  |
| 15-19 | 36 | 1.17 | 640 | 4.65 |
| 20-24 | 367 | 11.88 | 2 645 | 19.23 |
| 25-29 | 1 165 | 37.70 | 5 314 | 38.63 |
| 30-34 | 1 101 | 35.70 | 3 791 | 27.56 |
| 35-39 | 368 | 11.92 | 1 198 | 8.71 |
| >40 | 51 | 1.65 | 167 | 1.21 |
| **Maternal smoking in pregnancy** |  |  |  |  |
| Yes | 351 | 11.37 | 2 400 | 19.86 |
| No | 2 737 | 88.63 | 9 684 | 80.14 |
| **Parity** |  |  |  |  |
| 0 | 1 601 | 51.85 | 5 710 | 44.70 |
| 1 | 1 068 | 34.59 | 4 484 | 35.10 |
| 2 | 338 | 10.95 | 1 830 | 14.33 |
| 3 | 69 | 2.23 | 531 | 4.16 |
| 4+ | 12 | 0.39 | 219 | 1.72 |
|  |  |  |  |  |
| **Social class** |  |  |  |  |
| I | 258 | 8.35 | 590 | 5.90 |
| II | 1 128 | 36.53 | 3 150 | 31.48 |
| III (non-manual) | 1 235 | 39.99 | 4 281 | 42.79 |
| III (manual) | 190 | 6.15 | 783 | 7.83 |
| IV | 240 | 7.77 | 983 | 9.83 |
| V | 37 | 1.20 | 218 | 2.18 |
| **Maternal BMI** |  |  |  |  |
| Underweight (<18.5 kg/m2) | 138 | 4.47 | 569 | 4.97 |
| Normal (18.5-24.9) | 2 423 | 78.47 | 8 519 | 74.43 |
| Overweight (25-29.9) | 400 | 12.95 | 1 724 | 15.06 |
| Obese (≥30) | 127 | 4.11 | 633 | 5.53 |
| **GA (weeks)** | 3088 | 39.5 (1.7) | 13 755 | 39.4 (1.9) |
| **PTB** | 160 | 5.18 | 841 | 6.11 |
| **Birthweight (mean g)** | 3088 | 3410 (526) | 13 580 | 3392 (560) |
| **LBW** | 146 | 4.73 | 738 | 5.43 |
| **Age at scan (mean)** | 3088 | 17.8 (0.4) | 4149 | 17.8 (0.4) |
| **Gender** |  |  |  |  |
| Male | 1 416 | 45.85 | 7 105 | 51.65 |

Maternal and offspring characteristics for 3088 maternal-offspring pairs included in this analysis, and 13755 participants from the original ALSPAC sample with live offspring at one year and HDP data.

HDP, hypertensive disorder of pregnancy; GA, gestational age; PTB, preterm birth; LBW, low birth weight; BMD, bone mineral density

**Supporting Table 2: Associations between hypertensive disorders in pregnancy and offspring bone mineral density measured at 17**

|  |  |  |  |  |  |
| --- | --- | --- | --- | --- | --- |
| **Outcome** | **Model** | **Hypertensive disorder category** | **Mean difference** | **95% CI** | **p value** |
|  |  |  |  |  |  |
| **Total body** | **1** | **No HDP** | Ref |  |  |
|  |  | **Gesthyp** | 0.13 | 0.03, 0.22 | 0.007 |
|  |  | **PE** | -0.15 | -0.39, 0.08 | 0.191 |
|  | **2** | **No HDP** | Ref |  |  |
|  |  | **Gesthyp** | 0.13 | 0.04, 0.23 | 0.006 |
|  |  | **PE** | -0.14 | -0.37, 0.09 | 0.239 |
|  | **3** | **No HDP** | Ref |  |  |
|  |  | **Gesthyp** | 0.02 | -0.06, 0.10 | 0.595 |
|  |  | **PE** | -0.17 | -0.36, 0.02 | 0.075 |
|  | **4** | **No HDP** | Ref |  |  |
|  |  | **Gesthyp** | 0.01 | -0.06, 0.09 | 0.725 |
|  |  | **PE** | -0.18 | -0.37, 0.01 | 0.063 |
| **Spine BMD** | **1** | **No HDP** | Ref |  |  |
|  |  | **Gesthyp** | 0.19 | 0.09, 0.28 | <0.001 |
|  |  | **PE** | 0.04 | -0.20, 0.28 | 0.758 |
|  | **2** | **No HDP** | Ref |  |  |
|  |  | **Gesthyp** | 0.18 | 0.09, 0.28 | <0.001 |
|  |  | **PE** | 0.04 | -0.21,0.28 | 0.769 |
|  | **3** | **No HDP** | Ref |  |  |
|  |  | **Gesthyp** | 0.04 | -0.04, 0.12 | 0.372 |
|  |  | **PE** | -0.11 | -0.30, 0.09 | 0.295 |
|  | **4** | **No HDP** | Ref |  |  |
|  |  | **Gesthyp** | 0.03 | -0.05, 0.11 | 0.453 |
|  |  | **PE** | -0.11 | -0.31, 0.09 | 0.267 |
| **Total hip BMD** | **1** | **No HDP** | Ref |  |  |
|  |  | **Gesthyp** | 0.12 | 0.02, 0.21 | 0.017 |
|  |  | **PE** | -0.31 | -0.54, -0.07 | 0.011 |
|  | **2** | **No HDP** | Ref |  |  |
|  |  | **Gesthyp** | 0.13 | 0.03, 0.22 | 0.009 |
|  |  | **PE** | -0.29 | -0.52, -0.05 | 0.017 |
|  | **3** | **No HDP** | Ref |  |  |
|  |  | **Gesthyp** | 0.04 | -0.04, 0.12 | 0.344 |
|  |  | **PE** | -0.30 | -0.50, -0.10 | 0.004 |
|  | **4** | **No HDP** | Ref |  |  |
|  |  | **Gesthyp** | 0.03 | -0.05, 0.11 | 0.461 |
|  |  | **PE** | -0.30 | -0.50, -0.10 | 0.004 |

Table shows associations between hypertensive disorders of pregnancy and total body, spine and total hip BMD at 17, for 3088 maternal-offspring pairs included in the study*.* Model 1: age at scan & gender; Model 2: 1+maternal smoking, socioeconomic status, maternal age, parity; Model 3:2 + maternal BMI, offspring fat mass, lean mass and height; Model 4: 3 + birthweight & gestational age

***Supporting Table 3: Associations between hypertensive disorders in pregnancy and offspring bone mineral density measured at 17 with adjustment for calcium supplementation during pregnancy***

|  |  |  |  |  |  |
| --- | --- | --- | --- | --- | --- |
| **Outcome** | **Model** | **Hypertensive disorder category** | **Mean difference** | **95% CI** | **p value** |
|  |  |  |  |  |  |
| **Total body** | **1** | **No HDP** | Ref |  |  |
|  |  | **Gesthyp** | 0.13 | 0.03, 0.22 | 0.008 |
|  |  | **PE** | -0.09 | -0.33, 0.14 | 0.443 |
|  | **2** | **No HDP** | Ref |  |  |
|  |  | **Gesthyp** | 0.13 | 0.04, 0.23 | 0.006 |
|  |  | **PE** | -0.08 | -0.32, 0.16 | 0.495 |
|  | **3** | **No HDP** | Ref |  |  |
|  |  | **Gesthyp** | 0.02 | -0.05,0.10 | 0.567 |
|  |  | **PE** | -0.12 | -0.31, 0.07 | 0.228 |
|  | **4** | **No HDP** | Ref |  |  |
|  |  | **Gesthyp** | 0.02 | -0.06, 0.09 | 0.693 |
|  |  | **PE** | -0.13 | -0.32, 0.07 | 0.194 |
| **Spine BMD** | **1** | **No HDP** | Ref |  |  |
|  |  | **Gesthyp** | 0.18 | 0.09, 0.28 | 0.000 |
|  |  | **PE** | 0.10 | -0.15, 0.35 | 0.421 |
|  | **2** | **No HDP** | Ref |  |  |
|  |  | **Gesthyp** | 0.18 | 0.08, 0.28 | 0.000 |
|  |  | **PE** | 0.10 | -0.15, 0.35 | 0.442 |
|  | **3** | **No HDP** | Ref |  |  |
|  |  | **Gesthyp** | 0.04 | -0.04, 0.12 | 0.348 |
|  |  | **PE** | -0.05 | -0.25, 0.15 | 0.627 |
|  | **4** | **No HDP** | Ref |  |  |
|  |  | **Gesthyp** | 0.03 | -0.05, 0.11 | 0.424 |
|  |  | **PE** | -0.06 | -0.26, 0.15 | 0.579 |
| **Total hip BMD** | **1** | **No HDP** | Ref |  |  |
|  |  | **Gesthyp** | 0.12 | 0.02, 0.21 | 0.018 |
|  |  | **PE** | -0.27 | -0.51, -0.03 | 0.026 |
|  | **2** | **No HDP** | Ref |  |  |
|  |  | **Gesthyp** | 0.13 | 0.03, 0.22 | 0.010 |
|  |  | **PE** | -0.26 | -0.50, -0.02 | 0.034 |
|  | **3** | **No HDP** | Ref |  |  |
|  |  | **Gesthyp** | 0.04 | -0.04, 0.12 | 0.341 |
|  |  | **PE** | -0.28 | -0.48, -0.07 | 0.009 |
|  | **4** | **No HDP** | Ref |  |  |
|  |  | **Gesthyp** | 0.03 | -0.05, 0.11 | 0.458 |
|  |  | **PE** | -0.28 | -0.49, -0.07 | 0.009 |

Table shows associations between hypertensive disorders of pregnancy and total body, spine and total hip BMD, for 3075 maternal-offspring pairs*.* Model 1: age at scan & gender; Model 2: 1+maternal smoking, socioeconomic status, maternal age, parity, calcium supplementation; Model 3:2 + maternal BMI, offspring fat mass, lean mass and height; Model 4: 3 + birthweight & gestational age

**Supporting Table 4:** **Associations between hypertensive disorders in pregnancy and offspring bone mineral density measured at 9**

| **Outcome** | **Model** | **Hypertensive disorder category** | **Mean difference** | **95% CI** | **p value** |
| --- | --- | --- | --- | --- | --- |
| **Total body** | **1** | **No HDP** | Ref |  |  |
|  |  | **Gesthyp** | 0.06 | 0.02, 0.14 | 0.161 |
|  |  | **PE** | 0.09 | 0.12, 0.30 | 0.400 |
|  | **2** | **No HDP** | Ref |  |  |
|  |  | **Gesthyp** | 0.05 | -0.03, 0.14 | 0.216 |
|  |  | **PE** | 0.08 | -0.13, 0.30 | 0.436 |
|  | **3** | **No HDP** | Ref |  |  |
|  |  | **Gesthyp** | -0.02 | -0.07, 0.03 | 0.493 |
|  |  | **PE** | 0.10 | -0.02, 0.22 | 0.110 |
|  | **4** | **No HDP** | Ref |  |  |
|  |  | **Gesthyp** | -0.03 | -0.08, 0.02 | 0.236 |
|  |  | **PE** | 0.08 | -0.05, 0.20 | 0.218 |
| **Spine BMD** | **1** | **No HDP** | Ref |  |  |
|  |  | **Gesthyp** | 0.06 | -0.02, 0.15 | 0.148 |
|  |  | **PE** | 0.10 | -0.11, 0.32 | 0.341 |
|  | **2** | **No HDP** | Ref |  |  |
|  |  | **Gesthyp** | 0.06 | -0.03, 0.14 | 0.200 |
|  |  | **PE** | 0.10 | -0.13, -0.06 | 0.376 |
|  | **3** | **No HDP** | Ref |  |  |
|  |  | **Gesthyp** | -0.02 | -0.06, 0.03 | 0.553 |
|  |  | **PE** | 0.11 | -0.01, 0.24 | 0.082 |
|  | **4** | **No HDP** | Ref |  |  |
|  |  | **Gesthyp** | -0.03 | -0.08, 0.02 | 0.280 |
|  |  | **PE** | 0.09 | -0.04, 0.21 | 0.161 |

Table shows associations between hypertensive disorders of pregnancy and total body and spine BMD, for 4290 maternal-offspring pairs*.* Model 1: age at scan & gender; Model 2: 1+maternal smoking, socioeconomic status, maternal age, parity; Model 3:2 + maternal BMI, offspring fat mass, lean mass and height; Model 4: 3 + birthweight & gestational age
